# Interpersonal synchrony when singing in a choir

**DOI:** 10.3389/fpsyg.2022.1087517

**Published:** 2023-01-11

**Authors:** Julia A. M. Delius, Viktor Müller

**Affiliations:** Center for Lifespan Psychology, Max Planck Institute for Human Development, Berlin, Germany

**Keywords:** singing, choir, coordination dynamics, synchronization, coupling, network topology dynamics

## Abstract

Singing in a choir has long been known to enhance well-being and protect mental health. Clearly, the experience of a uniquely harmonious social activity is very satisfying for the singers. How might this come about? One of the important factors positively associated with well-being is interpersonal action coordination allowing the choir to function as a whole. This review focuses on temporal coordination dynamics of physiological systems and/or subsystems forming part or the core of the functional substrate of choir singing. These coordination dynamics will be evaluated with respect to the concept of a superordinate system, or superorganism, based on the principles of self-organization and circular causality. We conclude that choral singing is a dynamic process requiring tight interpersonal action coordination that is characterized by coupled physiological systems and specific network topology dynamics, representing a potent biomarker for social interaction.

## 1. Introduction

Singing in a choir is a deeply enjoyable joint activity involving a group of people making music together, usually in different voices. It is well known to enhance the singers’ well-being in various samples of laypeople (Stewart and Lonsdale, 2016; Boyd et al., 2020; Pentikäinen et al., 2021; Campbell et al., 2022). A beneficial effect of a randomly assigned intervention of weekly choral singing on cognitive performance has been observed in a group of elderly people at high risk of dementia (Feng et al., 2020). Depressive symptoms in people with dementia in residential care were also shown to be mitigated by recreational choir singing (Baker et al., 2022). Given the crossover in neural networks between singing, speech and language, group singing is also thought to improve speech and voice skills in people with Parkinson’s disease and functional communication skills in patients with post-stroke aphasia and dementia (Monroe et al., 2020). It is also a culturally ancient practice among humans all over the world (Janata and Parsons, 2013; Elmer, 2021).

In the last decade, there has been increasing interest in the way joint activity is coordinated. Any kind of social interaction between individuals such as dancing, talking to each other, or playing a team sport or music requires exquisitely precise coordination within and across the participants in the interaction (Lindenberger et al., 2009; Sänger et al., 2012; Müller et al., 2013; Filho et al., 2016; Pérez et al., 2017; Ahn et al., 2018; Müller et al., 2018; Chauvigné et al., 2019; Basso et al., 2021; Dell’Anna et al., 2021). How is this achieved in the case of choral singing where many different aspects need to be adapted to each other? Not only does the rhythm and timing of a song need to be synchronized in real time, the tone and melody sung by each member of the group also needs to match in order for the song to be pleasing to the ear of the listener and to the singers themselves (Kirsh et al., 2013; Vickhoff et al., 2013; Bernardi et al., 2017). Moreover, different subsystems (e.g., respiratory, cardiac, vocalizing, etc.) are synchronized within and between the singers and the conductor, forming a common hyper-system space or superorganism that functions as a whole (Müller et al., 2018, 2019, 2021; Müller, 2022). In this review article, we provide a brief overview of (1) synchronization patterns and complex networks emerging during choir singing, (2) neural mechanisms of choral singing, and (3) the functioning of a choir as a superordinate system or superorganism. Finally, future directions for research are discussed.

## 2. Synchronization patterns and complex networks emerging during choir singing

**Figure 1:**
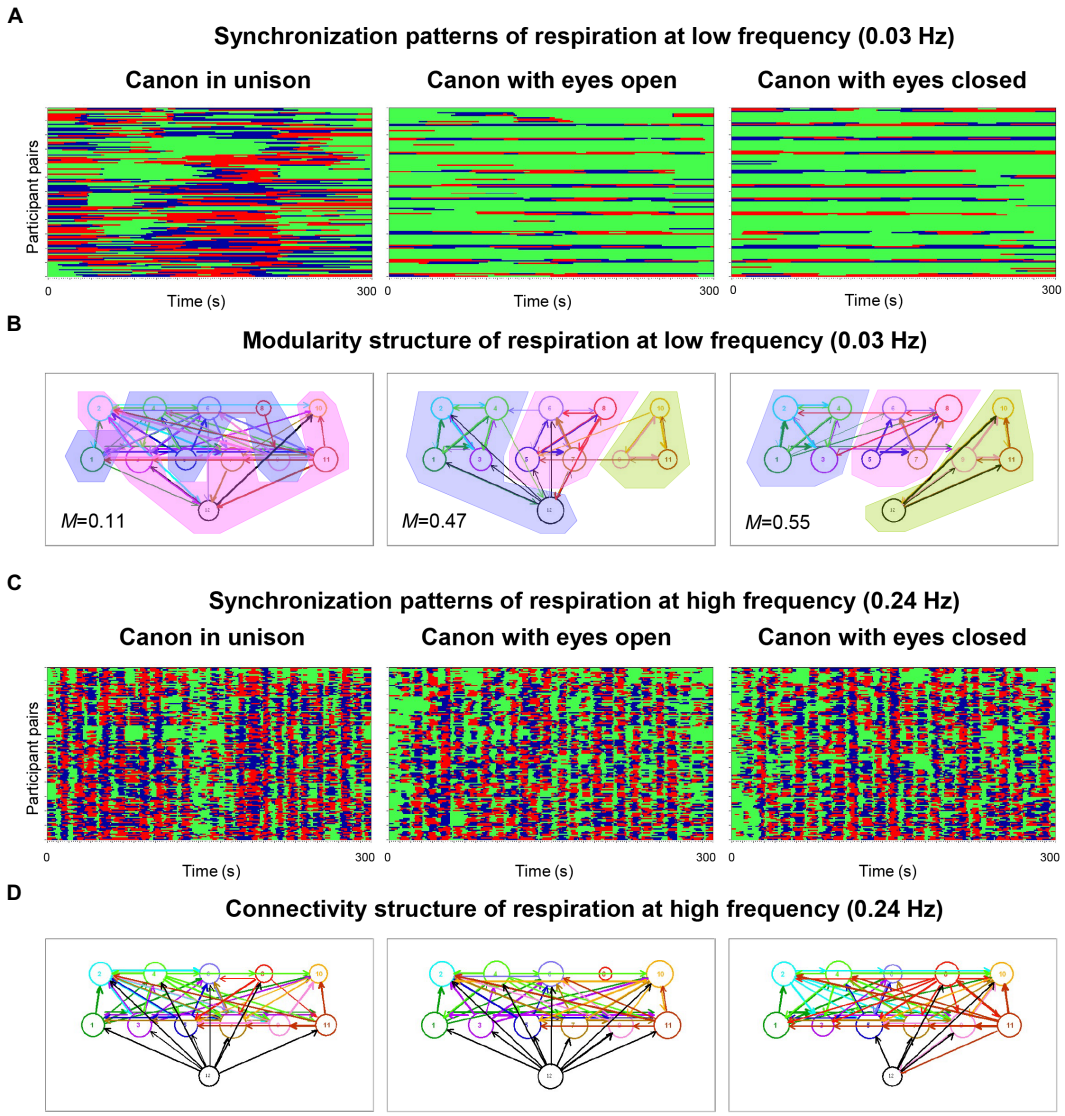
Phase synchronization patterns of the choir for respiration and corresponding modularity and connectivity structures. **(A)** Phase synchronization patterns at low respiration frequency (0.03 Hz). Pair-wise phase synchronization patterns of the whole choir (132 lines) for the three canon-singing conditions (300 s duration each): canon singing in unison (left column), canon singing with eyes open (middle column), and canon singing with eyes closed (right column). Each line represents one pair of subjects. For each subject, 11 lines represent the coded phase difference (Δφ) between this subject and all other choir members: –π/4 < Δφ < 0, blue stripes; 0 < Δφ < + π/4, red stripes; Δφ < −π/4 or Δφ > + π/4, green stripes or non-synchronization. High phase synchronization occurred when the choir sang the canon in unison. **(B)** Modularity structure of respiration at low frequency (0.03 Hz). Diagrams display directed networks for the Integrative Coupling Index (ICI) determined for the three canon-singing conditions (as in A). The size of the circle representing choir participants depends on the number of both incoming and outgoing connections. The thickness of the links corresponds to the connection strength, and the arrow displays the direction of the causal influence. The colored areas display the partition of the networks into modules or communities (*M* indicates modularity value), which are strongly related to different groups based on the parts sung in the choir. When the conductor sang along with the third entry during the singing of the canon with eyes closed, she was unambiguously assigned to this group. In the case of canon singing with eyes open, the conductor was partitioned into group 1, probably because she spent the most time singing with this group. Note that the modularity during canon singing in unison was low and the partition into modules is therefore blurred. **(C)** Phase synchronization patterns at high respiration frequency (0.24 Hz). Pair-wise phase synchronization patterns of the whole choir represented as in A. Again, high phase synchronization occurred when singing canon in unison rather than when singing with multiple voices. **(D)** Connectivity structure of respiration at high frequency (0.24 Hz). Diagrams display directed networks based on the ICI determined for the three canon-singing conditions (as in B). The connections of the conductor to the choir are predominantly directed from the conductor to the choir members, indicating that changes in respiration patterns or phases occur in the conductor earlier than in the choir members and are then transmitted from the conductor to the choir (adapted with permission from Müller and Lindenberger, 2011).

In pioneering work on physiological systems’ synchronization when singing in a choir, Müller and Lindenberger (2011) simultaneously measured the respiration and the electrocardiogram (ECG) of the 11 singers and the conductor of a choir of laypeople. The study found that phase synchronization of respiration and cardiac responses at six frequencies of interest (0.03, 0.05, 0.08, 0.11, 0.16, and 0.24 Hz) was higher during singing than during silent rest and generally higher when singing in unison than when singing a canon or song with multiple voice parts. Figure 1A shows this relationship for respiration at low frequency (0.03 Hz) under the three canon-singing conditions. Phase synchronization patterns indicate that some participants tended to be early in their phase course (marked red), whereas others tended to be late (marked blue); for some participants, the phase courses were mixed. Interestingly, as shown in Figure 1B, the network based on the respiratory coupling (at this frequency) between choir members when singing different parts of a canon could be partitioned into different modules or communities in accordance with the parts sung, but not when singing these in unison. Importantly, this partitioning worked preferably at low frequencies. Phase synchronization patterns at a high oscillation frequency (0.24 Hz), as shown in Figure 1C, indicate different phase difference patterns (like Figure 1A) that result in differences in the directed coupling measure used in this study (Integrative Coupling Index, *ICI*; Müller and Lindenberger, 2011). Most importantly, the *ICI* at 0.24 Hz showed strong, mostly unidirectional influences of the conductor on the choir members (see Figure 1D), indicating that changes in the oscillatory activity of respiration (and also heart rate variability, HRV) occurred in the conductor before the choir members, in accordance with the conductor’s functional role in the choir. Basic outcomes were confirmed in a recently published hyperscanning study that used the same synchronization indices for respiration and HRV in eight *professional* singers (Lange et al., 2022). The authors not only extended those findings to a non-homophonic musical repertoire, but also revealed an increase in synchronization of respiration during choral singing with *physical contact* that was significant across different frequency ranges. The effect of physical contact was stronger when all singers in the choir were singing in comparison to the partial ensemble singing.

In a different design, Vickhoff et al. (2013) examined respiratory sinus arrhythmia in a group of 18-year-olds humming, singing a hymn, or a mantra together and showed that song structure, respiration, and heart rate are connected. That is, at baseline (silently reading a text), individuals’ HRV was not synchronized and did not reveal a dominant frequency. During humming, each singer exhibited a dominant HRV frequency, but only when they sang a hymn or a mantra together did these frequencies align, with the highly structured mantra eliciting a frequency of 0.1 Hz for all singers. In a follow-up experiment, they examined five other subjects in the same conditions and focused on respiratory sinus arrhythmia, which was strongest in the 0.1 Hz frequency during singing of the mantra. Singing thus produces slow, regular, and deep respiration, which in turn triggers respiratory sinus arrhythmia that is associated with vagal influence. Vickhoff et al. (2013) propose that the interaction between parasympathetic and sympathetic activity during singing elicit the subjective well-being that singers report. Ruiz-Blais et al. (2020) followed up these ideas by examining dyadic vocalization among individuals without previous choral experience while measuring participants’ respiration and their ECG. Rather than singing a song together, participant dyads were given four different tasks: (1) breathing in synchrony, (2) synchronizing short notes, (3) singing notes of long duration and synchronizing the beginnings and ends of the note, and (4) singing out-of-phase short notes without singing together. The authors found that synchronization of respiration mediated HRV coupling when non-experts vocalized together. Whereas HRV coupling was mainly driven by synchronization of the respiratory activity (*cf.* Schäfer et al., 1998), joint vocalization contributed to it beyond the effect of respiration (Ruiz-Blais et al., 2020). A different example of dyadic interaction involving singing was investigated by Markova et al. (2020) who observed dyadic gaze synchrony as well as affect synchrony in mothers singing to their infants. In a review, Markova et al. (2019) suggested that infant-directed singing may serve the specific function to establish interpersonal synchrony as very young infants already respond to the temporal organization of musical sequences and older infants are more engaged when listening to singing than to speech.

Hemakom et al. (2017) proposed a new method to analyze coupled nonlinear and non-stationary multivariate signals, namely intrinsic synchrosqueezing transform (ISC), and used it to analyze respiration and HRV in two choirs. The first experiment assessed the respiration and ECG of three bass singers in a choir of 18 people in 4 min and 40 s periods of a low-stress rehearsal and a high-stress public performance and showed that the algorithm was able to detect highly localized synchrony in both types of signals. In another experiment, singer subsets of a 20-member choir (soprano and bass singers) performing for 1 h were investigated. It was found that the levels of cooperation/synchrony for both respiration and HRV markedly increased during most of the songs and decreased during the long pauses.

Palmer et al. (2019) took a different approach by examining endogenous rhythms, auditory and visual cues as well as body movement in experienced singers who sang solo and in duets in unison and in a canon condition, facing toward each other and away from each other. They found that the more similar the spontaneous (and temporally unprescribed) tempo of the solo singing, the less asynchronous the duet performances were, indicating that more similar natural frequencies make it easier for singers to coordinate. It was also evident that they used visual signals to coordinate: the duets were more synchronous when they were facing each other and they used head bobs to signal the rhythm, with the respective singer leading the canon marking time more strongly. Music performance thus requires a strong association between timing and movement. The role of visual contact between singers has also been pointed out in other studies (e.g., D’Amario et al., 2018, 2019a).

**Figure 2:**
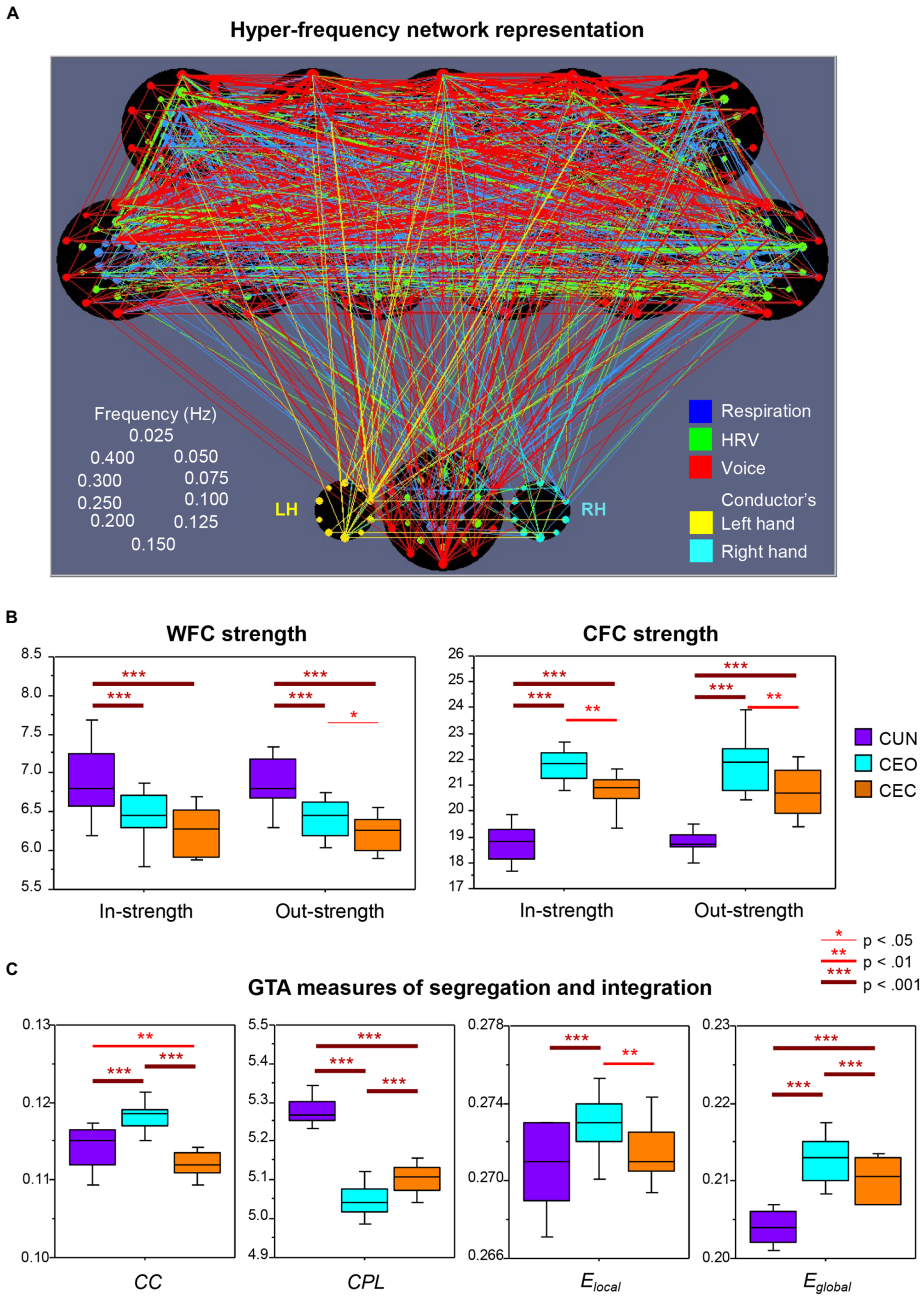
Representation and properties of the hyper-frequency choir network. **(A)** Representation of the hyper-frequency choir network. Choir network with 11 choir members (top) and the conductor (bottom); each choir member contains 30 nodes for three subsystems (coded by color) and 10 frequencies (ordered clockwise beginning from the top; see schema on the left). The conductor also yields 10 frequency nodes for the left and the right hand (LH and RH), correspondingly. The different subsystems are color-coded: respiration, blue; heart rate variability, green; voice, red;FIGURE 2 (Continued)conductor’s hand movement, yellow (see legends on the right). Each node’s circle size reflects that node’s out-strength. The connectivity strength between the nodes is indicated by the width of the line, and the color corresponds to the outgoing link. Note that the choir singing the canon in parts with eyes open is presented here. **(B)** Within- and cross-frequency coupling strengths. Within-frequency coupling (WFC) and cross-frequency coupling (CFC) in- and out-strengths under the three canon conditions: CUN, canon singing in unison; CEO, canon singing with eyes open; and CEC, canon singing with eyes closed. In- and out-strength are the sum of all weighted incoming and outgoing connections, respectively. Note the significantly highest WFC strength when the canon is sung in unison and the significantly highest CFC strength when it is sung in parts. **(C)** Graph-theoretical approach (GTA) measures of segregation and integration. Changes of segregation and integration GTA measures across the three canon conditions (see legend in B): *CC*, clustering coefficient; *CPL*, characteristic path length; *E_local_*, local efficiency; and *E_global_*, global efficiency. Note the significantly highest segregation (*CC* and *E_local_*) and integration (*E_global_* and shortest *CPL*) of the choir hyper-frequency network when singing the canon in parts with eyes open (CEO; adapted with permission from Müller et al., 2018, 2019).

In a follow-up analysis of the dataset used by Müller and Lindenberger (2011); Müller et al. (2018) also included the vocal audio signals and the conductor’s hand movements and expanded the analysis by considering both within-frequency coupling (WFC) and cross-frequency coupling (CFC) conjointly, using a newly developed network construction method that integrates all subsystems and frequency components into a common network. They showed that besides the respiratory and cardiac subsystems, vocalizing patterns and hand movement oscillations of the choir’s conductor also synchronized with one another during singing. Figure 2A displays the connectivity structure of this complex choir hyper-frequency network (HFN). It can be seen that the choir members and the conductor are strongly interconnected across the subsystems. Interestingly, the right hand of the conductor is strongly influenced by her left hand (unidirectional coupling). Furthermore, the connections between the conductor’s left hand and the choir members are mostly outgoing (i.e., from the hand to the choir), whereas the right hand contains both in- and out-going connections. This result is less surprising if we take into account that the conductor was left-handed. Thus, the unidirectional coupling from the left hand indicates its dominance and leading functional role related to control of the choir. The conductor’s other subsystems, especially respiration and voice production, also play a crucial role in the choral network organization (*cf.* Müller and Lindenberger, 2011). The tendency to precede or lag behind a co-performer was also found in the study with electrolaryngography and acoustic analysis to detect the onset and offset of phonation as well as the beginning and ending of notes in duet singers (D’Amario et al., 2019b). A further interesting result of the study by Müller et al. (2018), as shown in Figure 2B, is the significantly highest WFC when the choir sang the canon in unison and the significantly highest CFC when they sang the canon in parts with eyes open. While the former result is in line with that reported earlier by Müller and Lindenberger (2011), the latter goes beyond the initial findings and indicates that the interaction and coordination of the different canon entries in the choir is apparently supported by using different oscillation frequencies and interactions between them (Müller et al., 2018). Moreover, as shown in a further follow-up analysis by Müller et al. (2019) and in Figure 2C, the hyper-frequency choir network is characterized by enhanced segregation, indicated by the highest clustering coefficient and local efficiency, as well as by enhanced integration, indicated by the shortest characteristic path length and the highest global efficiency, when singing the canon in parts with eyes open as compared to two other canon conditions. As indicated by the authors, “High segregation indicates that choir members build smaller clusters in the choir (e.g., canon groups singing different parts), while high integration can indicate that notwithstanding the high segregation of the choir, its members remain strongly connected to each other (e.g., attending to the singers in the other groups). The CFC connections apparently play a crucial role in this integration. High segregation and integration of the choir when singing the canon in parts also indicates that the choir HFN is a small-world network, especially in this condition” (Müller et al., 2019, p. 10). It should be noted that small-world networks, possessing unique processing or information transfer capabilities, are regarded as a universal property of natural systems that are both locally and globally efficient (Watts and Strogatz, 1998; Latora and Marchiori, 2001; Telesford et al., 2011).

Taken together, singing with others has been shown to elicit synchronization across the singers in various systems ranging from respiration, HRV, to vocalization and movement. This synchrony can be enhanced through visual or physical contact and is also dependent on the frequency and musical structure that can be best described in terms of complex networks or HFNs, demonstrating a robust interplay between network topology and function (*cf.* Bashan et al., 2012).

## 3. Neural mechanisms of choral singing

Relatively little has been published on the neural basis of singing in a choir. Where human singing is at the center of attention (rather than birdsong, which has attracted much interest; e.g., Elemans, 2014), the focus is usually on individual singing, and on its connection with speech. For example, Zarate (2013) gave a comprehensive review of the control processes necessary during singing. As well as the complex interaction of the musculature steering the opening of the larynx and changes of the vocal folds, the muscles supporting respiration such as the diaphragm and the thoracic and abdominal muscles, and the musculature of the mouth, lips, and the jaw need to be controlled. A recent neuroanatomic study examining marmosets, a monkey species with remarkable vocal skills including vocal turn taking, showed that premotor areas, ventral area 6 (area 6 V) and the supplementary motor area, in the frontal lobe are larger in marmosets than in macaques, whose vocal skills are far more rudimentary (Cerkevich et al., 2022). These areas are also involved in human vocalization (e.g., Loh et al., 2020).

No less important is the auditory feedback that ensures monitoring of the vocal pitch (*cf.* Daffern and D’Amario, 2022). The details of the neural networks that are responsible for vocal motor control have often been determined *via* case studies of impairment after brain lesions or brain stimulation during neurosurgery. The networks are closely linked to speech and involve areas such as the reticular formation, the anterior cingulate cortex, and periaqueductal gray, and primary motor cortex. However, Van Lancker Sidtis et al. (2021) revealed dissociations between pitch, rhythm, and timing in speech versus singing and suggested that talking and singing arise from disparate neurological systems. Another study also found that singing, more than humming (“intoned speaking”), showed additional right-lateralized activation of the superior temporal gyrus, inferior central operculum, and inferior frontal gyrus (Özdemir et al., 2006). In line with this, patients with non-fluent aphasia due to left hemisphere lesions are often able to sing the text of a song while they are unable to speak the same words. In one study, stimulation of the cingulate cortex during neurosurgery was shown to elicit involuntary singing of spoken language (Bujarski et al., 2019), and another study described ictal singing in a case of left frontal lobe epilepsy (Enatsu et al., 2011).

Auditory feedback during singing is processed *via* the ascending auditory pathway from the cochlea to the medial geniculate nucleus in the thalamus and on to primary and secondary auditory cortex, Heschl’s gyrus, the right superior temporal gyrus, planum polare, and planum temporale. It seems that anterior insula is the area that integrates sensory feedback. One study was able to show that playing the cello makes use of some of the same neuronal networks as singing, such as Heschl’s gyrus, anterior insula, and anterior cingulate cortex (Segado et al., 2018). In an auditory event-related potentials (ERP) study, healthy choir singers and nonsinger controls aged over 60 years were investigated when listening passively to sounds (Pentikäinen et al., 2022). The authors used two simple oddball conditions, in which the pitch or spatial location of the sounds was varied, and a complex oddball condition involving encoding of abstract regularities in combinations of both pitch and location features. They showed that in the simple pitch and location conditions, the choir singers had smaller N1 ERP responses compared to the control subjects, whereas in the complex condition, the choir singers revealed a larger mismatch negativity (MMN) than the controls, which also correlated with better performance in a verbal fluency test. As the N1 ERP component reflects automatic stimulus processing and MMN is an indicator of stimulus change or of a neural-mismatch process triggered by a rare deviant stimulus at the presence of a neural trace of the frequent standard stimulus (*cf.* Mueller et al., 2008), the authors concluded that regular choir singing is associated with both more effective adaptation to simple sound features and more enhanced encoding of complex auditory regularities (Pentikäinen et al., 2022). A study that may provide insight into the special case of choral singing investigated the role of laypeople’s ability to sing and to move to the beat of a rhythmic stimulus (Dalla Bella et al., 2015). In line with the vocal learning and synchronization hypothesis that posits that motor synchronization to auditory rhythms may have evolved as a byproduct of selection for vocal learning (Patel, 2008), accurate and precise singers were also more accurate and more consistent in tapping in time to the beat.

Osaka et al. (2015) used fNIRS-based hyperscanning to show that the brains of two people singing together interact dynamically. The two singers were instructed to sing a nursery rhyme alone, listen to their experiment partner sing it, and then to sing it together. In another condition, they only hummed the tune. The authors applied wavelet transform coherence analysis between the two interacting brains and showed a significant increase in the neural synchronization between homologous channels of the left inferior frontal cortex (containing Broca’s area, which is involved in speech) in both cooperative singing and humming whereas synchronization between those channels in the right inferior frontal cortex (attributed to a coordinated production of melody) only occurred in the cooperative humming condition.

A completely different aspect of singing together was recently assessed by Bowling et al. (2022), who examined levels of cortisol, oxytocin, and testosterone, affect, subjective social connectedness, as well as heart rate (as a measure of physical exertion) before and after choral singing in comparison to speaking together. The authors found decreases in the salivary concentrations of oxytocin and cortisol after singing, but lower decreases in levels of oxytocin after singing than after speaking together. Self-reported affect was shifted in a positive direction and subjective social connectedness was also higher after singing together (see also Keeler et al., 2015). The feeling of “flow” (Csikszentmihalyi, 1975) elicited by singing together is often emphasized in this context.

In summary, there is a lack of in-depth research on the neural basis of singing together, even though this is an ancient human social activity of great interest. However, this short review of neural mechanisms reveals a complex interplay of different systems and subsystems including control of different muscle groups, sensory and motor feedback loops, steering of respiratory and cardiac activities accompanied by changes in cortisol and oxytocin levels, and probably much more.

## 4. The choir as a superordinate system or superorganism

Most research work on superorganisms refers to the animal kingdom, namely colonies of bees, ants, termites, or other social insects/animals (Wheeler, 1911, 1926; Emerson, 1939; Detrain and Deneubourg, 2006; Trianni et al., 2011; Hoffecker, 2013). As stated by Eberl (2010, p. 451), “A superorganism is a living system of a superior degree of complexity, consisting of many organisms. It may be defined more generally as a ‘collection of agents that can act in concert to produce phenomena governed by the collective’. Examples of superorganisms include ants and termite societies.” The microbiome consisting of microorganisms symbiotically inhabiting the human gut has also been defined as a superorganism, or even the entire human being with the commensal microbes inhabiting the skin, gut, etc. (e.g., Kramer and Bressan, 2015). The immune system that shapes homeostasis with a multitude of microbes and symbionts has also been considered as a superorganism (Eberl, 2010). The term has also been applied to football teams, pelotons (Trenchard, 2015), but also to an entire society, with its societal systems as a cognitive architecture (Boik, 2021). In the *Annual Review of Entomology*, superorganisms were defined as being “so tightly integrated that they possess features analogous to those of single organisms, including collective cognition” (Sasaki and Pratt, 2018, p. 259). In the specific case of a choir singing together, this integration reveals itself in the close within- and cross-frequency synchronization of respiration, HRV, vocal audio signals, and gestures across the individual members of the choir (Müller et al., 2018, 2019). As the authors noted, “The network dynamics of each individual singer [were] likely to be influenced by a complex coordination or the function of the choir as a whole” (Müller et al., 2018, p. 100). To function as a whole or a superorganism, the choir should not only be highly integrated but also highly segregated, as shown by Müller et al. (2019) and in Figure 2C. The finding that a choir is characterized by high segregation as well as integration and by specific network topology dynamics, especially when singing with different voices, reveals a superior degree of complexity that is important for superorganismic organization and functioning (Tononi and Edelman, 1998; Eberl, 2010). This functioning is based on complicated interactions of different subsystems that are known to produce a dynamic equilibrium in the organism and superorganism. A system’s coordination dynamics are based on the principles of self-organization and circular causality (Detrain and Deneubourg, 2006; Müller et al., 2021; Müller, 2022; Schiavio et al., 2022). Self-organization, defined as a process of spontaneous order arising from local interactions between parts of an initially disordered system, is an emergent property of the system (Haken, 1983, 1984, 2006; Haken and Portugali, 2016, 2021). In line with Haken’s (1983) principles of synergetics, self-organization has two directions: the upward direction with a local-to-global causation and the downward direction with a global-to-local determination, while the parts of the system cause the behavior of the whole and the behavior of the whole also constrains the behavior of its parts according to a majority rule of circular causation (*cf.* Buzsáki, 2006). In the case of the choir, each singer contributes to the choral singing through his or her voice and influences the function of the choir as a whole (upward causation); at the same time, the choir effectively functions as a superordinate system, or superorganism, that imposes boundary conditions on each individual singer (downward causation). This causation (upward and downward) not only concerns the voices but also other subsystems (e.g., respiratory, cardiac, etc.) contributing to the superordinate system dynamics and choir functioning (Müller et al., 2018, 2019, 2021; Müller, 2022).

**Figure 3:**
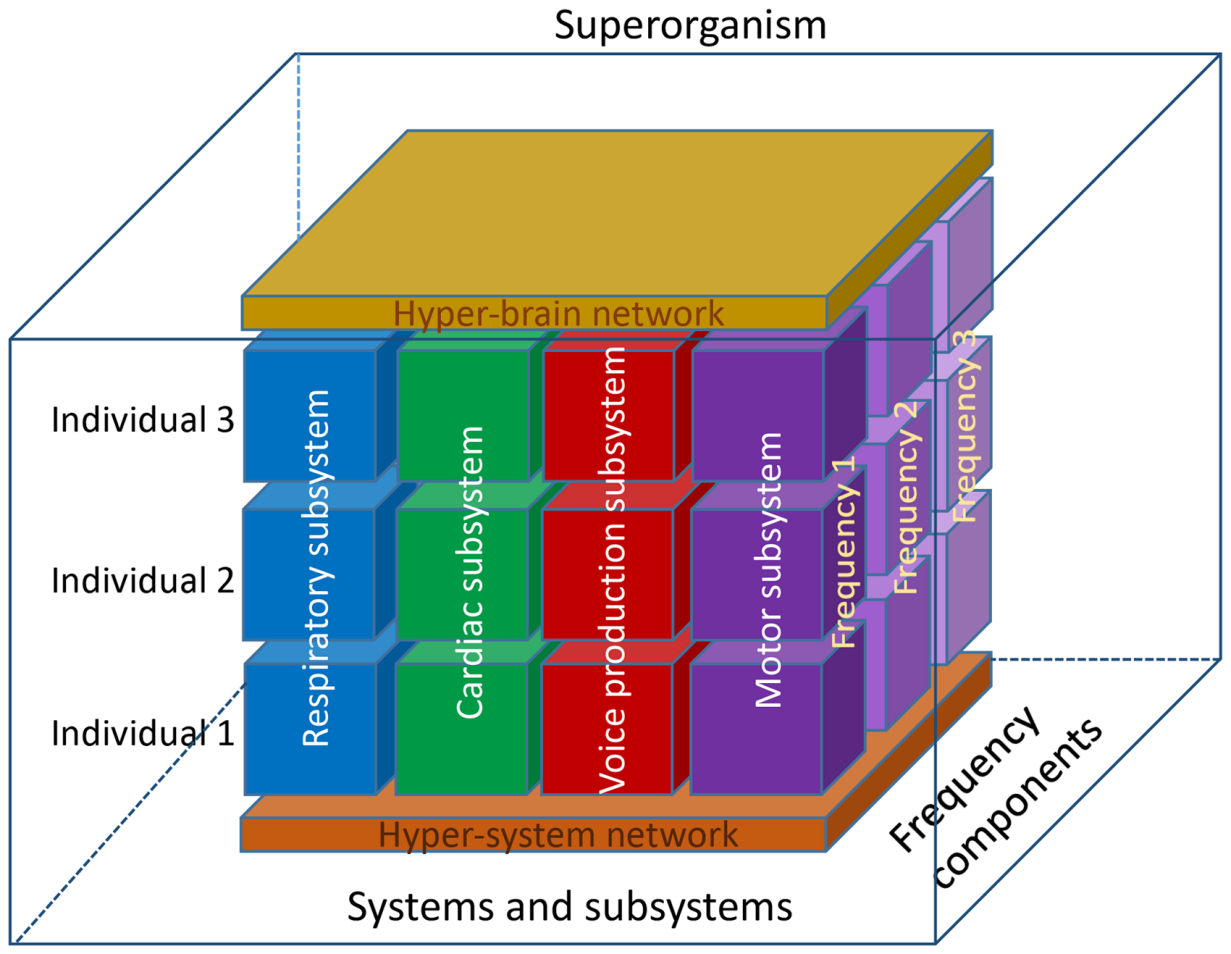
Schematic representation of the choir as a superorganism. This representation shows three individuals, four subsystems, and three oscillation frequency components. At the lower level, these components are organized into a hyper-system network, and at the higher level, into a hyper-brain network, which is also responsible for the neural control of different systems and subsystems. Together, these blocks with different types of connections at different levels and frequency components within and between the individuals represent a superorganism functioning as a whole. Note that this model represents only a limited number of components, which in reality may be much more. It is well known and has also been shown that auditory input or physical contact between choir members, for example, play an important role in choral singing. Therefore, the model can be further extended and include further components, but nevertheless the interplay between the different components (both homologous and crossover) would play a crucial role and provide the basis for a superordinate system or superorganism.

In this section, we took the perspective of the choir as a superorganism, a term usually associated with social insects forming a colony, but also with herds of mammals or with group adaptation (Detrain and Deneubourg, 2006; Gardner and Grafen, 2009). However, the synchronization occurring within and between the singers in various subsystems makes a compelling case for this interpretation of choral singing (see schematic representation of the choir as a superorganism in Figure 3).

## 5. Conclusion and future directions

Singing in a choir is a coordinated social interaction involving a group of people making music together, usually in different voices. This coordinated activity requires complex interaction between singers and their physiological systems and subsystems. In this review, we showed that systems and subsystems are strongly coupled to each other during singing, both within and between different frequencies and within and between singers. Due to these couplings, networks of different complexity levels can be formed and topological properties of these networks can be studied. The review shows how these properties can change depending on the singing conditions and which mechanisms can be assumed to be involved. We also reported on research outcomes about neural mechanisms of choral singing. Unfortunately, the literature is very scarce in this regard, especially that relating to multibrain activity of choral singing or hyperscanning. Nevertheless, several neural mechanisms have been identified that may play an important role in singing. Further (especially hyperscanning) studies are needed to better understand the neural mechanisms of joint singing. Finally, we tried to understand und represent choral singing as a superordinate system or superorganism that functions in accordance to a circular causation rule based on complementary upward and downward causality.

We are aware that this review is not able to cover the entire literature on choral singing and underlying neural and physiological mechanisms, but recognize that this growing field of research has certain knowledge gaps, making it difficult to provide an exhaustive view on these interesting phenomena. Clearly, further sophisticated research is needed to deepen our understanding.

## Author contributions

VM conceptualized the review. JD and VM wrote sections of the manuscript. Both authors contributed to the manuscript revision, read, and approved the submitted version.

## Funding

This research was funded by the Max Planck Society.

## Conflict of interest

The authors declare that the research was conducted in the absence of any commercial or financial relationships that could be construed as a potential conflict of interest.

## Publisher’s note

All claims expressed in this article are solely those of the authors and do not necessarily represent those of their affiliated organizations, or those of the publisher, the editors and the reviewers. Any product that may be evaluated in this article, or claim that may be made by its manufacturer, is not guaranteed or endorsed by the publisher.

## References

[ref1] Ahn S. Cho H. Kwon M. Kim K. Kwon H. Kim B. S.. (2018). Interbrain phase synchronization during turn-taking verbal interaction: a hyperscanning study using simultaneous EEG/MEG. Hum. Brain Mapp. 39, 171–188. doi: 10.1002/hbm.23834, PMID: 29024193 PMC6866597

[ref2] Baker F. A. Lee Y. E. C. Sousa T. V. Stretton-Smith P. A. Tamplin J. Sveinsdottir V.. (2022). Clinical effectiveness of music interventions for dementia and depression in elderly care (MIDDEL): Australian cohort of an international pragmatic cluster-randomised controlled trial. Lancet Healthy Longev. 3, e153–e165. doi: 10.1016/S2666-7568(22)00027-7, PMID: 36098290

[ref3] Bashan A. Bartsch R. P. Kantelhardt J. W. Havlin S. Ivanov P. C. (2012). Network physiology reveals relations between network topology and physiological function. Nat. Commun. 3:702. doi: 10.1038/ncomms1705, PMID: 22426223 PMC3518900

[ref4] Basso J. C. Satyal M. K. Rugh R. (2021). Dance on the brain: enhancing intra- and inter-brain synchrony. Front. Hum. Neurosci. 14:584312. doi: 10.3389/fnhum.2020.584312, PMID: 33505255 PMC7832346

[ref5] Bernardi N. F. Snow S. Peretz I. Perez H. D. O. Sabet-Kassouf N. Lehmann A. (2017). Cardiorespiratory optimization during improvised singing and toning. Sci. Rep. 7:8113. doi: 10.1038/s41598-017-07171-2, PMID: 28808334 PMC5556092

[ref6] Boik J. C. (2021). Science-driven societal transformation, part III: design. Sustainability. 13:726. doi: 10.3390/su13020726

[ref7] Bowling D. L. Gahr J. Graf Ancochea P. Hoeschele M. Canoine V. Fusani L.. (2022). Endogenous oxytocin, cortisol, and testosterone in response to group singing. Horm. Behav. 139:105105. doi: 10.1016/j.yhbeh.2021.105105, PMID: 34999566 PMC8915780

[ref8] Boyd M. von Ranson K. M. Whidden C. Frampton N. M. A. (2020). Short-term effects of group singing versus listening on mood and state self-esteem. Psychomusicology 30, 178–188. doi: 10.1037/pmu0000266

[ref9] Bujarski K. Martin C. Jobst B. Roberts D. Connolly A. (2019). Electrical stimulation of the cingulate elicits involuntary singing. Epileptic Disord. 21, 449–452. doi: 10.1684/epd.2019.1096, PMID: 31708490

[ref10] Buzsáki G. (2006). Rhythms of the Brain. Oxford: Oxford University Press.

[ref11] Campbell Q. Bodkin-Allen S. Swain N. (2022). Group singing improves both physical and psychological wellbeing in people with and without chronic health conditions: a narrative review. J. Health Psychol. 27, 1897–1912. doi: 10.1177/13591053211012778, PMID: 33913360

[ref12] Cerkevich C. M. Rathelot J.-A. Strick P. L. (2022). Cortical basis for skilled vocalization. Proc. Natl. Acad. Sci. U. S. A. 119:e2122345119. doi: 10.1073/pnas.2122345119, PMID: 35507879 PMC9171651

[ref13] Chauvigné L. A. S. Walton A. Richardson M. J. Brown S. (2019). Multi-person and multisensory synchronization during group dancing. Hum. Mov. Sci. 63, 199–208. doi: 10.1016/j.humov.2018.12.005, PMID: 30583090

[ref14] Csikszentmihalyi M. (1975). Play and intrinsic rewards. J. Humanist. Psychol. 15, 41–63. doi: 10.1177/002216787501500306

[ref15] D’Amario S. Daffern H. Bailes F. (2018). Synchronization in singing duo performances: the roles of visual contact and leadership instruction. Front. Psychol. 9:1208. doi: 10.3389/fpsyg.2018.01208, PMID: 30065685 PMC6057305

[ref16] D’Amario S. Daffern H. Bailes F. (2019a). Perception of synchronization in singing ensembles. PLoS One 14:e0218162. doi: 10.1371/journal.pone.0218162, PMID: 31188896 PMC6561579

[ref17] D’Amario S. Daffern H. Bailes F. (2019b). A new method of onset and offset detection in ensemble singing. Logop. Phoniatr. Vocology 44, 143–158. doi: 10.1080/14015439.2018.1452977, PMID: 29583026

[ref18] Daffern H. D’Amario S. (2022). “Understanding expressive ensemble singing through acoustics” in Together in Music. eds. Timmers R. Bailes F. Daffern H. (Oxford: Oxford University Press), 129–138.

[ref19] Dalla Bella S. Berkowska M. Sowiński J. (2015). Moving to the beat and singing are linked in humans. Front. Hum. Neurosci. 9:663. doi: 10.3389/fnhum.2015.00663, PMID: 26733370 PMC4683993

[ref20] Dell’Anna A. Leman M. Berti A. (2021). Musical interaction reveals music as embodied language. Front. Neurosci. 15:667838. doi: 10.3389/fnins.2021.667838, PMID: 34335155 PMC8317642

[ref21] Detrain C. Deneubourg J. L. (2006). Self-organized structures in a superorganism: do ants “behave” like molecules? Phys. Life Rev. 3, 162–187. doi: 10.1016/j.plrev.2006.07.001

[ref22] Eberl G. (2010). A new vision of immunity: homeostasis of the superorganism. Mucosal Immunol. 3, 450–460. doi: 10.1038/mi.2010.20, PMID: 20445502

[ref23] Elemans C. P. H. (2014). The singer and the song: the neuromechanics of avian sound production. Curr. Opin. Neurobiol. 28, 172–178. doi: 10.1016/j.conb.2014.07.022, PMID: 25171107

[ref24] Elmer S. S. (2021). Song transmission as a formal cultural practice. Front. Psychol. 12:654282. doi: 10.3389/fpsyg.2021.654282, PMID: 34955939 PMC8694211

[ref25] Emerson A. E. (1939). Social coordination and the superorganism. Am. Midl. Nat. 21, 182–209. doi: 10.2307/2420380

[ref26] Enatsu R. Hantus S. Gonzalez-Martinez J. Soab N. (2011). Ictal singing due to left frontal lobe epilepsy: a case report and review of the literature. Epilepsy Behav. 22, 404–406. doi: 10.1016/j.yebeh.2011.07.019, PMID: 21889414

[ref27] Feng L. Romero-Garcia R. Suckling J. Tan J. Larbi A. Cheah I.. (2020). Effects of choral singing versus health education on cognitive decline and aging: a randomized controlled trial. Aging 12, 24798–24816. doi: 10.18632/aging.202374, PMID: 33346748 PMC7803497

[ref28] Filho E. Bertollo M. Tamburro G. Schinaia L. Chatel-Goldman J. di Fronso S.. (2016). Hyperbrain features of team mental models within a juggling paradigm: a proof of concept. PeerJ. 4:e2457. doi: 10.7717/peerj.2457, PMID: 27688968 PMC5036110

[ref29] Gardner A. Grafen A. (2009). Capturing the superorganism: a formal theory of group adaptation. J. Evol. Biol. 22, 659–671. doi: 10.1111/j.1420-9101.2008.01681.x, PMID: 19210588

[ref30] Haken H. (1983). Synergetics: An Introduction – Nonequilibrium Phase Transitions and Self-Organization in Physics, Chemistry. Berlin: Springer.

[ref31] Haken H. (1984). The Science of Structure: Synergetics. New York: Van Nostrand Reinhold.

[ref32] Haken H. (2006). Information and Self-Organization: A Macroscopic Approach to Complex Systems. Berlin: Springer.

[ref33] Haken H. Portugali J. (2016). Information and selforganization: a unifying approach and applications. Entropy 18:197. doi: 10.3390/e18060197

[ref34] Haken H. Portugali J. (2021). Information and selforganization II: steady state and phase transition. Entropy 23:707. doi: 10.3390/e23060707, PMID: 34199648 PMC8229616

[ref35] Hemakom A. Powezka K. Goverdovsky V. Jaffer U. Mandic D. P. (2017). Quantifying team cooperation through intrinsic multi-scale measures: respiratory and cardiac synchronization in choir singers and surgical teams. Roy. Soc. Open Sci. 4:170853. doi: 10.1098/rsos.170853, PMID: 29308229 PMC5748960

[ref36] Hoffecker J. F. (2013). The information animal and the super-brain. J. Archaeol. Method Th. 20, 18–41. doi: 10.1007/s10816-011-9124-1

[ref37] Janata P. Parsons L. M. (2013). “Neural mechanisms of music, singing, and dancing” in Language, Music, and the Brain: A Mysterious Relationship. ed. Arbib M. A. (Boston, MA: MIT Press), 307–328.

[ref38] Keeler J. R. Roth E. A. Neuser B. L. Spitsbergen J. M. Waters D. J. Vianney J. M. (2015). The neurochemistry and social flow of singing: bonding and oxytocin. Front. Hum. Neurosci. 9:518. doi: 10.3389/fnhum.2015.00518, PMID: 26441614 PMC4585277

[ref39] Kirsh E. R. van Leer E. Phero H. J. Xie C. Khosla S. (2013). Factors associated with singers’ perceptions of choral singing well-being. J. Voice 27, 786.e25–786.e32. doi: 10.1016/j.jvoice.2013.06.004, PMID: 24119639

[ref40] Kramer P. Bressan P. (2015). Humans as superorganisms: how microbes, viruses, imprinted genes, and other selfish entities shape our behavior. Perspect. Psychol. Sci. 10, 464–481. doi: 10.1177/1745691615583131 26177948

[ref41] Lange E. B. Omigie D. Trenado C. Müller V. Wald-Fuhrmann M. Merrill J. (2022). In touch: cardiac and respiratory patterns synchronize during ensemble singing with physical contact. Front. Hum. Neurosci. 16:928563. doi: 10.3389/fnhum.2022.928563, PMID: 35992947 PMC9390082

[ref42] Latora V. Marchiori M. (2001). Efficient behavior of small-world networks. Phys. Rev. Lett. 87:198701. doi: 10.1103/PhysRevLett.87.198701 11690461

[ref43] Lindenberger U. Li S.-C. Gruber W. Mueller V. (2009). Brains swinging in concert: cortical phase synchronization while playing guitar. BMC Neurosci. 10:22. doi: 10.1186/1471-2202-10-22, PMID: 19292892 PMC2662862

[ref44] Loh K. K. Procyk E. Neveu R. Lamberton F. Hopkins W. D. Petrides M.. (2020). Cognitive control of orofacial motor and vocal responses in the ventrolateral and dorsomedial human frontal cortex. Proc. Natl. Acad. Sci. U. S. A. 117, 4994–5005. doi: 10.1073/pnas.1916459117, PMID: 32060124 PMC7060705

[ref45] Markova G. Nguyen T. Hoehl S. (2019). Neurobehavioral interpersonal synchrony in early development: the role of interactional rhythms. Front. Psychol. 10:2078. doi: 10.3389/fpsyg.2019.02078, PMID: 31620046 PMC6759699

[ref46] Markova G. Nguyen T. Schätz C. der Eccher M. (2020). Singing in tune – being in tune: relationship between maternal playful singing and interpersonal synchrony. Enfance 1, 89–107. doi: 10.3917/enf2.201.0089

[ref47] Monroe P. Halaki M. Kumfor F. Ballard K. J. (2020). The effects of choral singing on communication impairments in acquired brain injury: a systematic review. Int. J. Lang. Commun. Disord. 55, 303–319. doi: 10.1111/1460-6984.12527, PMID: 32096327

[ref48] Mueller V. Brehmer Y. von Oertzen T. Li S.-C. Lindenberger U. (2008). Electrophysiological correlates of selective attention: a lifespan comparison. BMC Neurosci. 9:18. doi: 10.1186/1471-2202-9-18, PMID: 18237433 PMC2270855

[ref49] Müller V. (2022). Neural synchrony and network dynamics in social interaction: a hyper-brain cell assembly hypothesis. Front. Hum. Neurosci. 16:848026. doi: 10.3389/fnhum.2022.848026, PMID: 35572007 PMC9101304

[ref50] Müller V. Delius J. A. M. Lindenberger U. (2018). Complex networks emerging during choir singing. Ann. N. Y. Acad. Sci. 1431, 85–101. doi: 10.1111/nyas.13940, PMID: 30058160

[ref51] Müller V. Delius J. A. M. Lindenberger U. (2019). Hyper-frequency network topology changes during choral singing. Front. Physiol. 10:207. doi: 10.3389/fphys.2019.00207, PMID: 30899229 PMC6416178

[ref52] Müller V. Lindenberger U. (2011). Cardiac and respiratory patterns synchronize between persons during choir singing. PLoS One 6:e24893. doi: 10.1371/journal.pone.0024893, PMID: 21957466 PMC3177845

[ref53] Müller V. Ohström K.-R. P. Lindenberger U. (2021). Interactive brains, social minds: neural and physiological mechanisms of interpersonal action coordination. Neurosci. Biobehav. R. 128, 661–677. doi: 10.1016/j.neubiorev.2021.07.017, PMID: 34273378

[ref54] Müller V. Sänger J. Lindenberger U. (2013). Intra- and inter-brain synchronization during musical improvisation on the guitar. PLoS One 8:e73852. doi: 10.1371/journal.pone.0073852, PMID: 24040094 PMC3769391

[ref55] Osaka N. Minamoto T. Yaoi K. Azuma M. Minamoto Shimada Y. Osaka M. (2015). How two brains make one synchronized mind in the inferior frontal cortex: fNIRS-based hyperscanning during cooperative singing. Front. Psychol. 6:1811. doi: 10.3389/fpsyg.2015.01811, PMID: 26635703 PMC4659897

[ref56] Özdemir E. Norton A. Schlaug G. (2006). Shared and distinct neural correlates of singing and speaking. NeuroImage 33, 628–635. doi: 10.1016/j.neuroimage.2006.07.013, PMID: 16956772

[ref57] Palmer C. Spidle F. Koopmans E. Schubert P. (2019). Ears, heads, and eyes: when singers synchronise. Q. J. Exper. Psychol. 72, 2272–2287. doi: 10.1177/1747021819833968, PMID: 30744490

[ref58] Patel A. D. (2008). Music, Language and the Brain. New York: Oxford University Press.

[ref59] Pentikäinen E. Kimppa L. Makkonen T. Putkonen M. Pitkäniemi A. Salakka I.. (2022). Benefits of choir singing on complex auditory encoding in the aging brain: an ERP study. Ann. N. Y. Acad. Sci. 1514, 82–92. doi: 10.1111/nyas.14789, PMID: 35596717

[ref60] Pentikäinen E. Pitkäniemi A. Siponkoski S. T. Jansson M. Louhivuori J. Johnson J. K.. (2021). Beneficial effects of choir singing on cognition and well-being of older adults: evidence from a cross-sectional study. PLoS One 16:e0245666. doi: 10.1371/journal.pone.0245666, PMID: 33534842 PMC7857631

[ref61] Pérez A. Carreiras M. Duñabeitia J. A. (2017). Brain-to-brain entrainment: EEG interbrain synchronization while speaking and listening. Sci. Rep. 7:4190. doi: 10.1038/s41598-017-04464-4, PMID: 28646190 PMC5482847

[ref62] Ruiz-Blais S. Orini M. Chew E. (2020). Heart rate variability synchronizes when non-experts vocalize together. Front. Physiol. 11:762. doi: 10.3389/fphys.2020.00762, PMID: 33013429 PMC7506073

[ref63] Sänger J. Müller V. Lindenberger U. (2012). Intra- and interbrain synchronization and network properties when playing guitar in duets. Front. Hum. Neurosci. 6:312. doi: 10.3389/fnhum.2012.00312, PMID: 23226120 PMC3509332

[ref64] Sasaki T. Pratt S. C. (2018). The psychology of superorganisms: collective decision making by insect societies. Annu. Rev. Entomol. 63, 259–275. doi: 10.1146/annurev-ento-020117-043249, PMID: 28977775

[ref65] Schäfer C. Rosenblum M. G. Kurths J. Abel H. H. (1998). Heartbeat synchronized with ventilation. Nature 392, 239–240. doi: 10.1038/32567 9521318

[ref66] Schiavio A. Maes P.-J. van der Schyff D. (2022). The dynamics of musical participation. Music. Sci. 26, 604–626. doi: 10.1177/1029864920988319, PMID: 36090466 PMC9449429

[ref67] Segado M. Hollinger A. Thibodeau J. Penhune V. Zatorre R. J. (2018). Partially overlapping brain networks for singing and cello playing. Front. Neurosci. 12:351. doi: 10.3389/fnins.2018.00351, PMID: 29892211 PMC5985323

[ref68] Stewart N. A. J. Lonsdale A. J. (2016). It’s better together: the psychological benefits of singing in a choir. Psychol. Music 44, 1240–1254. doi: 10.1177/0305735615624976

[ref69] Telesford Q. K. Joyce K. E. Hayasaka S. Burdette J. H. Laurienti P. J. (2011). The ubiquity of small-world networks. Brain Connect. 1, 367–375. doi: 10.1089/brain.2011.0038, PMID: 22432451 PMC3604768

[ref70] Tononi G. Edelman G. M. (1998). Consciousness and complexity. Science 282, 1846–1851. doi: 10.1126/science.282.5395.1846 9836628

[ref71] Trenchard H. (2015). The peloton superorganism and protocooperative behavior. Appl. Math. Comput. 270, 179–192. doi: 10.1016/j.amc.2015.08.006

[ref72] Trianni V. Tuci E. Passino K. M. Marshall J. A. R. (2011). Swarm cognition: an interdisciplinary approach to the study of self-organising biological collectives. Swarm Intell. 5, 3–18. doi: 10.1007/s11721-010-0050-8

[ref73] Van Lancker Sidtis D. Kim Y. Ahn J. S. Sidtis J. (2021). Do singing and talking arise from the same or different neurological systems? Dissociations of pitch, timing, and rhythm in two dysprosodic singers. Psychomusicology 31, 18–34. doi: 10.1037/pmu0000270

[ref74] Vickhoff B. Malmgren H. Åström R. Nyberg G. Ekström S.-R. Engwall M.. (2013). Music structure determines heart rate variability of singers. Front. Psychol. 4:334. doi: 10.3389/fpsyg.2013.00334, PMID: 23847555 PMC3705176

[ref75] Watts D. J. Strogatz S. H. (1998). Collective dynamics of “small-world” networks. Nature 393, 440–442. doi: 10.1038/30918 9623998

[ref76] Wheeler W. M. (1911). The ant-colony as an organism. J. Morphol. 22, 307–325. doi: 10.1002/jmor.1050220206

[ref77] Wheeler W. M. (1926). Emergent evolution and the social. Science 64, 433–440. doi: 10.1126/science.64.1662.433 17741942

[ref78] Zarate J. M. (2013). The neural control of singing. Front. Hum. Neurosci. 7:237. doi: 10.3389/fnhum.2013.00237, PMID: 23761746 PMC3669747

